# Exotic pets in Ireland: 2. Provision of veterinary services and perspectives of veterinary professionals’ on responsible ownership

**DOI:** 10.1186/s13620-021-00191-5

**Published:** 2021-05-04

**Authors:** Matt Goins, Alison J. Hanlon

**Affiliations:** School of Veterinary Medicine, University College Dublin, Dublin, Ireland

**Keywords:** Exotic pet, Responsible pet ownership, Veterinary services, Veterinary profession, Veterinary practitioner, Veterinary nurse, White lists, Animal welfare

## Abstract

**Background:**

There has been increasing concern expressed about the welfare of exotic pets worldwide. For the purposes of this article, an exotic pet is considered to be a non-domesticated species, where there are knowledge gaps on good practice (minimum standards of care), veterinary diagnostics and treatments. The categories of exotic pets included in this study were: small mammals (< 20 kg), large mammals (> 20 kg), birds, reptiles, amphibians, fish and invertebrates. Dogs, cats, rabbits, hamsters, gerbils, guinea pigs, mice, rats, and ferrets were excluded from the study. An online survey of veterinary professionals conducted between July and August 2020 provides the first empirical data for Ireland. In this pilot study (the second in this thematic series) we aim to characterise the provision of veterinary services to exotic pets from the veterinary professionals’ perspective, explore the main concerns of veterinary professionals towards exotic pet ownership, and their recommendations to support responsible exotic pet ownership.

**Results:**

Using an online survey this pilot study gathered evidence from 63 veterinary professionals currently working in private practice in Ireland. The prevalence of veterinary services for exotic pets in Ireland was determined to be 82% of small and mixed animal clinics of respondents’ practices ranging from 9.1 to 100% for different categories of exotic pets. The most common issues encountered in practice with exotic pets were related to nutrition, environment, and behaviour followed by clinical diseases such as respiratory, infectious, and gastrointestinal issues. The most common concerns veterinary professionals had with exotic pet ownership related to the lack of owner knowledge as well as the lack of veterinary knowledge and accessible resources. The most common strategic initiatives indicated by veterinary professionals included black or white lists (to prohibit and permit the keeping of exotic pet species respectively), licensing for owners and increased availability of CPD for veterinary professionals.

**Conclusions:**

More than four in every five veterinary professionals in small or mixed animal practices surveyed were willing to treat exotic pets and in many cases they already were. A scaffold for best practice is required to support the health and welfare of exotic pets and responsible ownership. Keystones include veterinary education to support veterinary professionals with daily practice, establishing a white list of exotic species that are suitable as pets, registration at the point of purchase to enable traceability for biosecurity purposes and research to identify care standards to support the health and welfare of exotic pets.

**Supplementary Information:**

The online version contains supplementary material available at 10.1186/s13620-021-00191-5.

## Background

As discussed in the first part of this thematic series exploring exotic pets in Ireland [1], there is no generally accepted definition of an exotic pet across national or international veterinary bodies [2–4] with differences in the definitions used by the BZVS [2] and AVMA [3] and the nomenclature between Europe and North America. For example, the use of the term ‘pocket pets’ which is used to refer to rodents, ferrets, and rabbits as well as animals such as hedgehogs and sugar gliders [5]. For the purposes of this pilot study, an exotic pet is considered to be a non-domesticated species, where there are knowledge gaps on good practice (minimum standards of care), veterinary diagnostics and treatments. The categories of exotic pets included in this study were: small mammals (< 20 kg), large mammals (> 20 kg), birds, reptiles, amphibians, fish and invertebrates. Dogs, cats, rabbits, hamsters, gerbils, guinea pigs, mice, rats, and ferrets were excluded from the study.

The prevalence of exotic pet ownership in the Republic of Ireland is unreported, however a pilot study of staff at University College Dublin in 2020 showed that 34.4% of respondents owned an exotic pet [1]. There are several concerns about exotic pet ownership regarding animal welfare, threats to biodiversity associated with exotic species being released into the wild and zoonotic risks. From a veterinary perspective, Whitehead [6] and Lennox [7] have noted that a key barrier to the provision of veterinary services for exotic pets is a lack of species-specific information in terms of routine diagnostic tests and treatments. Species-specific requirements such as drug dosages remain unvalidated, in practice dosages may be calculated on the basis of personal experience or “best guess” [6]. Furthermore Warwick [8] noted that many veterinary professionals were generally unaware of pathogenic agents in exotic species. These factors compromise the capacity of veterinary professionals to provide the same level of expertise as they do for common domestic pets such as dogs and cats.

The second part of this thematic series [1] focused on the experience of veterinary professionals regarding the provision of services for exotic pets and initiatives to support responsible exotic pet ownership. A key tenet of responsible pet ownership is to be able to provide for the animal needs and the challenge with exotic animals is that there are gaps in the evidence to support the health and welfare of exotic pets at both the owner level and in veterinary practice. At a governance level, one approach has been to identify species that are “*suited to being kept as companion animals*” [4] by creating black lists and white lists. Simply put white lists, also known as positive lists, are lists of species allowed to be kept as companion animals while black lists, also known as negative lists, are lists of species banned from being kept as companion animals [4]. A white list generally represents a more manageable, proportionate, and effective regulatory process with a much lower burden on enforcement agencies [4]. Currently, Belgium, the Netherlands, and Luxembourg [9] are the only countries in the EU operating white lists [4, 9, 10]. The white lists in Belgium and the Netherlands are currently restricted to mammal species [10, 11]. Luxembourg is the only EU member state operating a white list for exotic pet species [9]. Conversely 18 EU countries have black lists of some form [10]. However, black lists are generally seen as being much more cumbersome to enforce with exotic pet owners quickly finding their way around them [12]. For example, when the EU banned the importation of red eared sliders (a species of turtle), yellow eared sliders quickly became a popular alternative and were also released into the environment [12].

The Federation of Veterinarians of Europe (FVE) have even gone so far as to call for the creation of white lists across the EU [4], which takes on additional importance in the face of the COVID-19 pandemic with its likely zoonotic origin [13] as well as from a One Health perspective with the general knowledge that the majority of emerging infectious diseases worldwide are zoonotic [14]. Pets are also acknowledged to be a risk factor for illnesses caused by various pathogens [15] with the exotic pet trade producing several high profile invasive species [16]. Animal surveillance measures are a vital cog in the machine in the face of zoonotic risks. In Ireland the Animal Health and Welfare (Sale or Supply of Pet Animals) Regulations 2019 [17] require traceability from the point of origin to the point of sale to a private owner.

The aim of this pilot study was to characterise the provision of veterinary services for exotic pets in Ireland, explore the main concerns of veterinary professionals towards exotic pet ownership, and their recommendations to support responsible exotic pet ownership.

## Results

### Respondent demographics

A total of 63 individuals accessed the survey. All respondents consented to the survey, however two respondents did not answer any additional questions. Of the remaining 61 respondents, 17 were private veterinary practitioners, 43 were veterinary nurses, and one was a veterinary educator. Thirty-eight respondents worked in small animal practice, 20 in mixed animal practice, and three in “Other”. The three “Other” responses comprised a “mostly exotics” practice, a wildlife rescue practice, and the University College Dublin Veterinary Hospital.

Of the 61 respondents, eight identified as male and 53 identified as female. Thirty-eight graduated and received their qualification between 2011 and 2020, 17 between 2001 and 2010, and 6 between 1991 and 2000. Eighteen per cent of respondents owned an exotic pet, which is a lower rate than was found in the general public in part one of this pilot study [1]. Exotic pets that respondents reported owning included birds (8.2%), reptiles (9.8%), amphibians (1.6%), invertebrates (1.6%), small exotic mammals (3.3%), and large exotic mammals (1.6%). There was no significant difference between the prevalence of ownership between male and female veterinary professionals (*p* = 1.00) or the reported provision of veterinary services for exotic pets between mixed and small animal practices (*p* = 0.29). There was also no significant difference in the prevalence of exotic pet ownership between veterinary practitioners and nurses (*p* = 0.15) or the likelihood that a veterinary practitioner or nurse worked in a practice that treated exotic pets (*p* = 0.71).

### Provision of veterinary services for exotic pets

Overall, 82% of respondents said that their practice treated exotic animals. However, only 39 respondents (64%) answered questions about the provision of veterinary services for individual categories of exotic pets, ranging from 9.10% (amphibians) extending to 100% (birds) in mixed animal practices (see Table 1). There were no significant differences in the provision of veterinary services for different categories of exotic pets found between small animal and mixed animal practices.

**Table 1:** Prevalence of provision of veterinary services per exotic pet category reported by respondents in small animal practice or mixed animal practice

Exotic pet category	Percentage of small animal practices with exotic pet clients (n)	Percentage of mixed animal practices with exotic pet clients (n)
Small Mammals (< 20 kg)	89.3 (25)	72.7 (8)
Large Mammals (> 20 kg)	25.0 (7)	45.5 (5)
Birds	96.4 (27)	100 (11)
Reptiles	78.6 (22)	54.5 (6)
Fish	28.6 (8)	27.3 (3)
Amphibians	46.4 (13)	9.10 (1)
Invertebrates	32.1 (9)	18.2 (2)

The most common number of patients in a respondent’s practice per category of exotic pet was 1–10, birds were the most common exotic pet treated (100+) whilst small exotic mammals were the second most common for individual practices. Of practices with exotic pet clients, small exotic mammals and birds were generally seen most often (monthly), while other categories were seen less frequently (quarterly or annually), although one small animal practice reported seeing bird patients daily and another reported seeing both bird and small exotic mammal patients daily.

On the topic of the approach taken by veterinary practices for a consultation with an exotic pet where the respondent had no clinical experience in treating the species, the most common strategy was that a practice colleague with an interest in exotics would take the consultation (*n* = 18, 46% of respondents) followed by applying a first principles approach (*n* = 12, 30.77%) and referral of the patient to another practice (*n* = 5, 12.82%).

### Most common issues reported by owners

The most common issues encountered during consultations in veterinary practice were related to nutrition (*n* = 27, 69.23% of respondents), environment (*n* = 22, 56.41%), and behaviour (*n* = 7, 17.95%) followed by various medical issues such as respiratory, infectious, and gastrointestinal issues. One respondent noted that disease processes are generally far more advanced in exotic pets by the time they see them in practice compared with domestic pets.

### Most common issues in treating exotic pets by veterinary practitioners

The most common issues encountered in veterinary practice regarding exotic pet patients were the lack of in-house expertise in a particular species/category of exotic pet (*n* = 28, 71.79% of respondents), no suitable equipment with which to treat exotic pets (*n* = 27, 69.23%), and a lack of diagnostic tests available for these species (*n* = 17, 43.59%). Other notable responses included constrained budgets of owners for treatment, a lack of licensed drugs available to veterinary practitioners, and that veterinary practitioners were unable to provide the level of service expected by the client.

### Main concerns of exotic pet ownership and development of strategies to support improved health and welfare of exotic pets

By far the most common concerns veterinary professionals have with exotic pet ownership related to the lack of owner knowledge (*n* = 32, 82.05% of respondents) and the lack of veterinary knowledge and accessible resources (*n* = 17, 43.59%). Other concerns that were highlighted were the ease of purchase of exotic pets, the lack of legislation on ownership of exotic pets, perceptions that clients considered exotic pets to be disposable, and an unwillingness of owners to pay for treatment or care.

The most common policy recommendations suggested by veterinary professionals focused on regulations namely, black or white lists indicating which exotic species could be kept as pets and guidelines on the minimum conditions, breeding, and sale of exotics, ownership education programmes, and certification or licensing to own specific species. Furthermore, respondents indicated an interest in continuing professional development (CPD) courses including a mandatory requirement for CPD per year on exotic pet medicine.

On the topic of what organisation should take the lead role in implementing these strategies, the Department of Agriculture, Food and the Marine was the most commonly suggested organisation followed by the Veterinary Council of Ireland.

## Discussion

A key challenge of the survey was gaining access to veterinary professionals in Ireland due to data protection laws. Because of this, veterinary professionals in Ireland were contacted by the Irish Veterinary Nurses Association and via social media. As such, the response rate for the survey cannot be determined.

### Availability of veterinary services

This pilot study found that the majority of veterinary professionals from small and mixed animal practices treated exotic pet species, especially birds and reptiles. This is counter to the views expressed by respondents in the first part of this thematic series [1], which indicated poor availability of veterinary services. This disparity may reflect a difference in expectations between clients and veterinary professionals regarding the level of service or expertise available. For example, veterinary surgeons may adopt a first principles approach while pet owners may be seeking veterinary practitioners with postgraduate training in their pet’s species. This is consistent with a viewpoint expressed in the Veterinary Record [18], where a client’s choice of veterinary practice was determined by the type and severity of the clinical condition of their exotic pet. The author described retaining the service of a local veterinary practice with no specialist training in exotic pet medicine for emergency stabilisation and treatment of time-sensitive conditions, but travelled to a specialist practice (40 min journey) for other conditions [18].

### Most common issues seen in veterinary practice

Veterinary professionals’ concerns regarding the health and welfare of exotic pets largely mirrored those of the pet owners [1], namely the lack of information and materials on caring for exotic pets. An important caveat to this is that both groups detailed difficulty in either accessing or providing quality care. Veterinary professionals referred to the cost sensitivity of owners as a barrier to providing appropriate veterinary care with one particular respondent advising “*owner willingness to spend money*” was a main concern with exotic pet owners and in relation to owners providing “*finances to provide adequate husbandry and ongoing care*”. Similar findings have been reported in the US, which showed a reduced willingness to pay for the veterinary care of exotic pets, approximately 40% less than on dogs and 70% less than on cats [7]. Whilst longevity of some exotic pet species and costs of care may be less than those of traditional pets, these socio-economic differences could have detrimental consequences for the actual provision of care for exotic pets.

### Main concerns of exotic pet ownership and development of strategies

Similar to the findings of the first part of this thematic series [1] and supported by the literature [6–8, 19], this study found that the main concerns of veterinary professionals in the provision of veterinary care to exotic pets was a lack of available information, both for the owner and veterinary professional, as well as a lack of available resources, such as diagnostic tests, views which are consistent with recent publications from the UK [18, 20]. Respondents were also concerned with the ease with which owners could procure exotic pets and that these animals were perceived as disposable by some clients. This concern of disposability of certain types of exotic pets is reported in the literature [21], and corresponds to the low cost of purchase of certain pets such as budgies.

Suggested strategies and policy proposals to support the health and welfare of exotic pets included provision of exotic pet medicine focused CPD, ownership education programmes, certification/licensing to own specific species, and more strict consequences for violating animal welfare. Regulations on minimum conditions, breeding, and sale of exotics were also suggested as was the creation of either a white list (pertaining to those species allowed to kept as pets), such as those enacted by the Netherlands and Belgium for mammal species [4] and Luxembourg for mammal and exotic species [9], or a black list (prohibiting the keeping of those species that are unsuitable to be kept as pets) such as those in place in 18 other EU nations [10] or Florida State Statute Section 379.372, which regulates certain non-native invasive reptiles such as various python species including the Burmese python as well as multiple lizard species.

In this spirit, a scaffold to establish “best practice” for exotic pets in Ireland could begin with a white list and registration of ownership of exotic pets at the point of purchase. Registration is important to inform veterinary education and service provision, but most notably for biosecurity purposes. The COVID-19 pandemic has highlighted zoonotic risks, and with an estimated 75% of emerging infectious disease being zoonotic in origin [22], supporting traceability through a national database should be considered. Recent legislation in Ireland such as the Animal Health and Welfare (Sale or Supply of Pet Animals) Regulations 2019 [17] requires pet stores and pet sellers to register and provide traceability of their animals. This database could be used to register ownership at the point of purchase for the purposes of biosecurity and traceability to facilitate contact tracing of pet owners who are keeping potential reservoir species, in the event of a zoonotic outbreak. Regulatory controls on the disease risks of exotic pets are also exemplified by the Commission Implementing Decision (EU) 2018/320 contains animal health protection measures for the trade of salamanders to reduce the spread of *Batrachochytrium salamandrivorans* (Bsal) a pathogenic fungus that causes significant morbidity and mortality in wild and captive salamanders. Research on the health and welfare of exotic pets should form the final keystone of “best practice” to build a robust evidence base to support responsible exotic pet ownership and the provision of veterinary services.

## Conclusion

More than four in every five veterinary professionals from small or mixed animal practices surveyed indicated a readiness to treat exotic pets. In many cases, they were already providing these services for a minority of their client base. This has implications for veterinary education to support the veterinary community with providing services to the exotic pet owning community. Adequate provision of education, within the veterinary education curriculum and/or as readily obtainable CPD are required to address the concerns of veterinary professionals.

Policy issues with exotic pet ownership need to be considered. For example establishing a ‘white list’, indicating which non-domesticated species are permitted to be kept as pets as instituted by the governments of Belgium [4], the Netherlands [4], and Luxembourg [9]. A licensing system of exotic pet owners should also be considered.

## Methods

### Survey design

An online survey for veterinary professionals was designed to explore the prevalence of veterinary services for exotic pets in Ireland. The inclusion criteria for veterinary professionals were that they had to be a registered veterinary practitioner or veterinary nurse, working in private practice in the Republic of Ireland. The survey consisted of 13 questions, divided into three sections: information about the respondent and their practice, information pertaining to the exotic pet caseload at the respondent’s practice, and responsible pet ownership and national strategy.

Section one consisted of consent to participate in the pilot study and questions about the respondent such as their role in the veterinary profession (veterinary practitioner, veterinary nurse, veterinary educator/academic, or other). Those who selected “veterinary educator/academic” or “other” were permitted to complete the survey; however, their data was excluded from the analysis. Year of graduation, type of practice worked in (small, mixed, large, etc), gender, and personal exotic pet ownership were requested in this section.

Section two consisted of five questions asking the respondent to enumerate the types of exotic pets* accessing their veterinary practice (*categorised as: small exotic mammals (< 20 kg), large exotic mammals (> 20 kg), birds, reptiles, amphibians, fish, and invertebrates), and the frequency of access by exotic pet clients to their veterinary practice. Information pertaining to most common issues, challenges and approaches to treating, and confidence in treating exotic pets were also requested in this section.

Section three consisted of two questions asking the respondent about their main concerns regarding exotic pet ownership in Ireland as well as potential policy approaches to support the health and welfare of exotic pets.

The survey was published online using SurveyMonkey® and was open for responses from 15 July 2020 until 5 August 2020. An invitation to complete the survey was distributed by the Irish Veterinary Nurses Association. In addition, the survey was shared on social media via University College Dublin School of Veterinary Medicine Twitter (@ucdvetmed) and @AlisonHanlon and the University College Dublin Veterinary Hospital Facebook page (https://www.facebook.com/ucdvet/) and the Veterinary Voices Ireland Facebook page (https://www.facebook.com/groups/vetvoicesireland/). A copy of the survey can be found in the Supplementary Materials.

### Data handling and analysis

Data were exported from SurveyMonkey® into Microsoft Excel (2013). R version 3.6.1 and R Studio version 1.2.1335 were used for data cleaning and transformation, data visualisation, generating descriptive statistics, and for all statistical analyses. To test for statistically significant differences in the frequencies of responses between independent cohorts, a Pearson’s Chi-Squared test or Fisher’s exact test was conducted depending on sample size. Comparisons were made between small animal practices and mixed animal practices and between male and female veterinary professionals. The significance threshold for statistical analyses was *p* < 0.05.

## Supplementary Information

**Additional file 1:** Exotic Pet Survey - Veterinary Professionals.

## Data Availability

The data generated during this study are available from the corresponding author on reasonable request.

## Abbreviations

BZVS: British Veterinary Zoological Society
AVMA: American Veterinary Medical Association
FVE: Federation of Veterinarians of Europe
CPD: Continuing Professional Development
EU: European Union
