# Supplementary material for: Exotic pets in Ireland: 2. Provision of veterinary services and perspectives of veterinary professionals’ on responsible ownership
Source: Ir Vet J. 2021 May 4;74:13. doi: 10.1186/s13620-021-00191-5 (PMC8096126; doi:10.1186/s13620-021-00191-5)
Supplement: Supplementary file 1 — Additional file 1: Exotic Pet Survey - Veterinary Professionals. [file 13620_2021_191_MOESM1_ESM.pdf]

Exotic Pet Survey - Veterinary Professionals

Welcome to the Exotic Pet Survey!

Dear Veterinary Professional,
I am a veterinary student at UCD, conducting a Summer Research Award project with Associate Professor Alison Hanlon. The project focuses on exotic pet ownership in Ireland, common issues with exotic pets and access to veterinary care. Exotic pets are defined for the purposes of this study as a household animal/pet that is not a dog, cat, rabbit, hamster, gerbil, guinea pig, mouse, rat, or ferret.

The survey consists of 3 sections. Section One (6 questions) is about you and your veterinary clinic, Section Two (5 questions) asks questions pertaining to your clinic's exotic pet caseload and Section 3 (2 questions) focuses on responsible pet ownership and national strategy. The survey should take < 10 minutes to complete. We would really appreciate your help us gain valuable insight into exotic pet ownership in Ireland.

The survey closes on 5 August 2020.

Sincerely,
Matt Goins 15200869@ucdconnect.ie

1. I give my consent to participate in this project and understand that the data will be used for research purposes and to support the development of veterinary education.

- ○ Agree
- ○ Disagree

Exotic Pet Survey - Veterinary Professionals

About You & Your Veterinary Practice

* 2. What is your role in the veterinary professional community in Ireland?

- ◯ Private veterinary practitioner
- ◯ Veterinary nurse
- ◯ Veterinary educator/academic
- ◯ Other e.g. veterinary researcher, student (please specify)

* 3. What type of practice are you?

- ◯ Small animal
- ◯ Large animal
- ◯ Mixed
- ◯ Other (please specify)

* 4. What year did you qualify (MVB, BScVN)

5. What is your gender

- ◯ Female
- ◯ Male
- ◯ Prefer not to say
- ◯ Other (please specify)

6. Do you own an exotic pet e.g. reptiles?

- ◯ Yes
- ◯ No
- ◯ If YES, what type of exotic pet do you own?

* 7. Does your clinic have clients with exotic pets?

- Yes
- No

Exotic Pet Survey - Veterinary Professionals

Type & Range of Exotic Pet Patients, & Common Issues

* 8. Please estimate the type and number of exotic species and frequency of consultations in your veterinary practice

	Approx. number of patients	Approx number of consultations per year (per category of exotic and not per client)
Small exotic mammals e.g. chinchilla		
Large exotic mammals e.g. pot bellied pig		
Birds		
Reptiles		
Fish		
Amphibians		
Invertebrates		

Other (please specify)

* 9. What are the most common issues seen with exotic pets in your practice? Tick all that apply.

- [ ] Behavioural issues
- [ ] Nutritional issues
- [ ] Environmental-related e.g. inappropriate temperature gradient
- [ ] Other health-related conditions (please specify)

* 10. What are the main problems with providing veterinary services for exotic pets? Tick all that apply.

- [ ] There are few diagnostic tests available
- [ ] We do not have suitable equipment for exotics in our practice
- [ ] Lack of in-house expertise
- [ ] Other (please specify)

11. What approach is taken in your practice for exotic species that you have no clinical experience in treating?

- ○ A practice colleague with an interest in exotics takes the consultation
- ○ It is referred to another practice
- ○ A first principles approach is adopted
- ○ Other (please specify)

12. On a scale of 1-10 please rate your confidence (10=high) in providing veterinary services for the following groups of exotic species.

Birds	
Reptiles	
Amphibians	
Small exotics mammals (<20kg)	
Large exotic mammals (>20kg)	
Invertebrates	

Responsible Pet Ownership & National Policy

* 13. What are your 3 main concerns regarding exotic pet ownership in Ireland?

1

2

3

14. In your opinion, what national policy approach is required to support the health and welfare of exotic pets? Who should take the lead role in developing the approach?

National policy:

Lead role:

Exotic Pet Survey - Veterinary Professionals

Thank-you!

We’d like to thank you again for taking part in this project. If you are interested to find out more about UCD research on animal welfare, please go to: www.ucd.ie/animalwelfare
